# Evaluation of Lymphorrhea and Incidence of Lymphoceles: 4DryField® PH in Radical Retropubic Prostatectomy

**DOI:** 10.1155/2016/2367432

**Published:** 2016-06-23

**Authors:** Johannes-J. Karsch, Martin Berthold, Jürgen Breul

**Affiliations:** Division of Urology, Loretto-Krankenhaus, Mercystraße 6-14, 79100 Freiburg, Germany

## Abstract

*Purpose*. To investigate impact of polysaccharide hemostat 4DryField PH (4DF) applied on lymph node dissection area after radical retropubic prostatectomy (RRP) on lymphorrhea and lymphocele (LC) formation.* Methods*. 104 consecutive patients underwent RRP, 51 without 4DF treatment (CT-group) and 53 with 4DF treatment (4DF-group). Groups were comparable (age, risk profile, and lymph node numbers). Postoperative drain loss (PDL) and development of early and late LC were analyzed (mean follow-up at 7 months: 100%).* Results*. PDL was 452.5 ± 634.2 mL without and 308.5 ± 214 mL with 4DF treatment. PDL > 1000 mL only occurred in CT-group (5/51). Overall, 45 LC (26 in CT- versus 19 in the 4DF-group) were diagnosed. At day 8, LC were equally distributed between groups. Incidence of late LC, however, was twice in controls (16/51) versus 4DF-patients (8/53). Symptomatic LC (4 in untreated patients, 2 in 4DF-patients) were treated with percutaneous drainage (duration: 45 days in untreated patients versus 12 days in 4DF-patients).* Conclusion*. Application of 4DF on lymph node dissection areas lessened total drain loss and significantly lowered high volume drain loss. Furthermore, 4DF reduced frequency of late lymphoceles and lymphoceles requiring treatment by half, as well as duration of percutaneous drainage by more than two-thirds.

## 1. Introduction

Lymphoceles (LC) are collections of lymphatic fluid resulting from leakage of afferent lymphatic vessels as it occurs due to tissue trauma or surgery. Although the incidence of LC can be reduced by meticulous surgical technique and careful sealing of lymph vessels [1, 2], LC are still a common complication following radical retropubic prostatectomy (RRP) [2–6]. In the literature, the incidence of asymptomatic LC varies between 30 and 60% [7–10] and about half of all secondary interventions after RRP are due to LC [4, 11, 12]. The development of symptomatic LC after RRP is associated with distinct impairment and consecutive costs [13–16]. Possible complications include pain and subsequent problems like infection with formation of abscesses, peripheral edema, thrombosis, and/or thromboembolism [17–22]. Furthermore, lymphadenectomy (LA) in RRP might be associated with oozing in delicate areas, in which the autonomic innervation of the lesser pelvis is present. Since conservative measures such as electrocautery, clipping, and ligation are somewhat traumatic and might cause nerval and vascular injury, the use of an atraumatic hemostat may be preferable.

**Figure 1:**
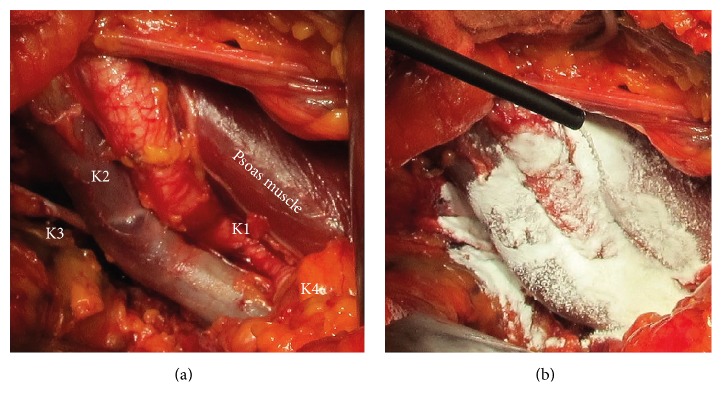
Site at the end of the surgical procedure after radical retropubic prostatectomy: (a) exposed iliac vessels after lymphadenectomy, (b) with polysaccharide application.

Since 2013 we use the polysaccharide based hemostatic agent 4DryField PH (4DF) from PlantTec Medical GmbH, Bad Bevensen, in the delicate area of the iliac artery and* fossa obturatoria* after RRP with LA to achieve low trauma, gentle hemostasis in patients with diffuse bleeding. This product is a modified polysaccharide of plant-based origin, CE-certified for hemostasis and adhesion prevention. Its hemostatic effect originates from its capability to rapidly absorb water, which accelerates the extrinsic coagulation cascade [23]. Additionally, when this polysaccharide powder is transformed into a gel by using 0.9% saline solution, it has the capability to provide adhesion prevention [24]. Since pelvic leg-lymph has a similar composition as plasma, it was conceivable for us that the polysaccharide might have not only a hemostatic but also a lymphostatic effect and, thus, might have an impact on LC formation [25–27]. In hemostasis, polysaccharide powders act as hydrophilic molecular sieves that immediately absorb the fluid blood components and concentrate blood solids such as platelets, red blood cells, and blood proteins, thereby accelerating the natural blood-clotting cascade [28–31]. A figure showing the mechanism of how polysaccharides assist in hemostasis can be found, for example, in Humphreys et al. [28, Figure 1] or Tschan et al. [29, Figure 1]. Although lymph does not contain platelets, it contains other clotting factors and exhibits coagulation mechanisms that are, although slower, generally comparable to those of blood [25, 32–37]. Therefore, it can be assumed that polysaccharides might have a similar effect in lymphostasis as in hemostasis.

The aim of the present prospective observational study was to compare patients consecutively treated with 4DF versus a similar group operated within the same period of time without polysaccharide treatment with respect to hemo- and lymphostasis as well as incidence of LC.

## 2. Materials and Methods

The areas of lymph node dissection are illustrated in Figure 1(a). Thereby, compartment K1 comprises the area from the femoral canal to remove the node of Cloquet to the bifurcation of the common iliac artery and medially from the middle external iliac vein to the genitofemoral nerve laterally, K2 from the middle external iliac vein to the obturator nerve, K3 beyond the obturator nerve, and K4 along the internal iliac artery. In case of limited dissection lymph nodes were removed from area K2, while in patients with extended dissection lymph nodes were erased from all areas. 53 of these patients had treatment with 4DF (4DF-group), 51 patients had no polysaccharide application and served as a control group (CT-group). Clips and ligation were used at the proximal and distal limits of the nodal packet; the dissection in the specific areas was done separately. All patients were operated on at the Department of Urology and Urological Oncology of the Loretto Hospital in Freiburg. All surgeons who performed the surgeries were experienced in conducting RRP with LA. Ambulatory or stationary rehabilitation took place mainly in the same external facilities and included continence training and medical controls such as ultrasonic evaluation. Stationary rehabilitation also comprised psychooncologic support. Extended lymph node dissection as described above was performed when preoperative analysis revealed 3 or more positive punch biopsies, PSA-value above 14, and a Gleason-Score of at least 4 + 3 = 7b. The ratio of patients with extended versus limited dissection was similar in both groups. In the 4DF-group, the polysaccharide was applied on the whole area of lymph node dissection as described above.  Figure 1(b) indicates how the polysaccharide was applied.

All data were collected and analyzed in a pseudonymized manner. Age, weight, size, concomitant diseases, stage of the prostate cancer, and number of dissected lymph nodes were evaluated. Furthermore, amount and duration of drain loss, early postoperative incidence, and volume of LC as determined with sonography on postoperative day 8 were evaluated during hospitalization following a standard institutional protocol. Drain tubes were removed after secretion had ceased completely. A blinded and experienced operator performed assessment of LC. Imaging after radical retropubic prostatectomy was conducted on postoperative day 8 before discharge of the patient. This timing of imaging was also employed by, for example, Schoeppler et al. [38]. Outpatient care reports (stationary and ambulatory rehabilitation) and/or telephone survey among doctors/hospitals were reviewed for incidence and/or persistence of LC. Furthermore, the duration of treatment of the LC that required intervention was evaluated. In this study LC treatment was done with percutaneous drains. Follow-up at an average of seven months postoperatively was 100%.

Data are displayed as group-median or group-mean with standard deviation. Statistical differences were evaluated using student's unpaired *t*-test performed with GraphPad Prism 6 (La Jolla, USA). Significance was assumed at *p* < 0.05. The time period of percutaneous drainage intervention for LC treatment could not be tested statistically due to the low case numbers of treated LC (CT-group: 4, 4DF-group: 2).

## 3. Results

This study includes 104 men at the age of 50 to 82 years (mean age 67 ± 7.5 years) in whom RRP with LA was performed between June 2013 and July 2014.  Table 1 summarizes clinical data and indicates that there were no significant differences in age, BMI, the number of resected lymph nodes, or duration of operation between patients with and without polysaccharide treatment. Gleason-Scores and median PSA-values can be found in Table 2.

### 3.1. Drain Output

**Figure 2:**
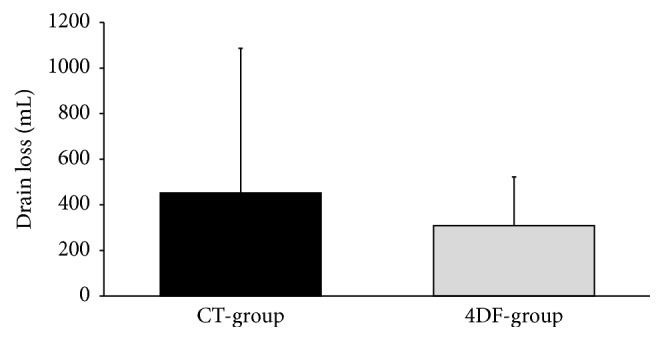
Drain loss (mean) of CT-group versus 4DF-group.

Application of 4DF on oozing areas resulted in effective hemostasis as confirmed by individual judgment of surgeons. The period of drainage did not differ significantly between the CT-group and the 4DF-group (3.1 ± 1.9 versus 2.8 ± 1.6 days; *p* = 0.41). Mean drain loss (Figure 2) was 452.5 ± 634.2 mL in CT-patients versus 308.5 ± 214 mL in 4DF-patients, not significantly different (*p* = 0.1062). However, five patients in the CT-group revealed a drain loss >1000 mL whereas this was not observed in 4DF-patients. The substantial drain losses of CT-patients are expressed in a distinctly higher standard deviation (Figure 2).  Table 3 indicates the impact of involvement of lymph nodes on amount of drain loss and LC development. Involvement of lymph nodes was not associated with increased drain loss or elevated incidence of LC.

### 3.2. Incidence of Early Lymphoceles

Eight days after surgery the incidence and volume of early LC were evaluated with sonography. In the CT-group 9 of 51 and in 4DF-group 9 of 53 patients revealed LC. Eight CT-patients had 1 and one patient had 2 LC. The volumes ranged from 8 to 45 mL (median 20 ± 13.5 mL). In the 4DF-group, 7 patients revealed 1 LC and 2 patients had 2 LC. Here the volumes ranged from 10 to 80 mL (median 19 ± 26 mL). Accordingly, in the CT-group, 10 LC were noted, and in 4DF-group, 11 LC were noted. This piece of data indicates that there was no difference with respect to number and size of early LC between both groups.

### 3.3. Incidence of Late Lymphoceles

Evaluation of late LC development was taken from the reports of stationary and ambulatory rehabilitation, providing sonographic data at median of 21 (10–62) days postoperatively. In the CT-group the total number of patients with LC increased from 8 to 12 patients. In only 3 of these patients LC had been diagnosed at postoperative day 8 sonography. This means that in 5 of the patients the early LC had disappeared, while there were 9 new patients with late LC in whom LC had not been detected early. Since 4 of these patients had developed 2 LC, a total of 16 late LC were recorded. Median volume of these LC was significantly higher than that of LC at postoperative day 8 (20 ± 13.5 mL versus 81.5 ± 98.7 mL; *p* = 0.0225).

In the 4DF-group, the total number of patients with LC had decreased from 9 to 8. Of the 8 patients with late LC, 2 had had LC at postoperative day 8. This means that 6 new patients had late LC in whom LC had not been detected early. Since none of the patients had developed more than 1 LC, a total of 8 late LC were recorded. The LC volumes (median) had increased significantly from 19 ± 26 mL at day 8 to 70 ± 56.9 mL at late sonography (*p* = 0.0042).

**Figure 3:**
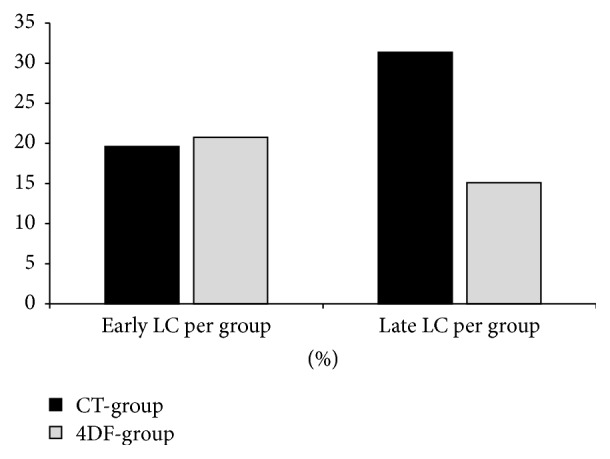
Early and late lymphocele findings as a percentage of the respective patient cohort size (CT-group: 51 patients, 4DF-group: 53 patients).

Figure 3 shows the total numbers of early and late LC in both groups. The total number of late LC in CT-patients (*n* = 16) was twice as high as that in 4DF-patients (*n* = 8).

### 3.4. Treatment of Lymphoceles

**Figure 4:**
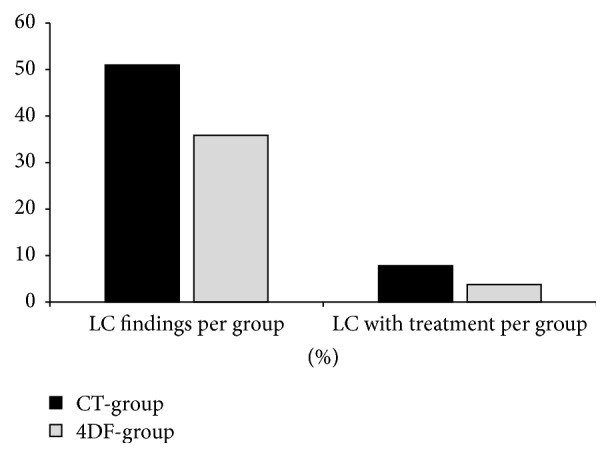
Total number of lymphoceles and number of lymphoceles with treatment, both as a percentage of the respective patient cohort size (CT-group: 51 patients, 4DF-group: 53 patients).

In 33 of 104 patients (31.7%), LC were detected. These 33 patients had a total of 45 LC. The incidence of LC in the CT-group (26 LC in 18 of 51 patients) was distinctly higher than in the 4DF-group (19 LC in 15 of 53 patients).  Figure 4 shows the total numbers of LC in both groups and the numbers of LC that were treated. Six patients (5.8% of all patients) necessitated LC treatment; four of them were CT-patients and two 4DF-patients.  Table 4 indicates complications leading to LC treatment, that is, vascular compression in 3 patients, pain in 2 patients, and edema in 1 patient.  Table 5 summarizes LC volume and drain period, incidence of early and late LC, and duration of treatment for both groups.

In 3 patients of the CT-group, additional sclerotherapy with doxycycline was performed without positive effect. Average treatment time was 44.5 ± 13.2 days in the CT-group and 12 ± 2.8 days in the 4DF-group. Accordingly, treatment time was 3.7 times longer in the CT-group as compared to the 4DF-group. After treatment there was no recurrence of LC up to a mean observation period of 7 months. None of the patients treated with the polysaccharide revealed adverse events that could be related to the product.

## 4. Discussion

Lymphadenectomy (LA) in radical retropubic prostatectomy (RRP) might be associated with oozing in delicate areas, in which the autonomic nerves are present. Since conventional measures like ligation, clipping, or electrocoagulation are associated with local trauma, we have used 4DF for diffuse bleeding from these areas and the intraoperative subjective surgeons' judgment confirmed efficiency. Another favorable aspect for the use of polysaccharide hemostats is their rapid degradation, which occurs within days. The good tolerability of 4DF, reported earlier [31, 39, 40], was confirmed in our study, since no side effects were observed.

The reduced incidence of high volume drain loss with polysaccharide treatment might also be a result of improved coagulation of lymph fluid resulting from unavoidable transection of lymph vessels in the course of LA. It is known that limb lymph, which is the major component of iliac lymph, contains coagulation factors similar to blood plasma and coagulates via the extrinsic coagulation pathway, similar to that described for hemostasis in* in vitro* experiments by Hanke et al. [23].

Furthermore, our observations indicate that application of the polysaccharide might have an impact on LC formation after RRP. This still is a common complication associated with possible problems such as pain, infection, and thrombosis/thromboembolism. About 50% of postoperative interventions not due to dysfunctional problems are due to LC [20, 41]. The incidence of asymptomatic LC varies between 30 and 60% [7–10], whereby most of the studies were performed on patients with only limited LA [4, 9]. The high variation might possibly be due to the use of different methods of detection and different time points of examination. In the present study, the overall incidence of LC formation was in the lower range as compared to the literature.

To reduce incidence of LC, Augustin et al. suggested that an implementation of pelvic LA should be considered carefully [4, 6]. In their study on 1243 patients operated on from 1999 to 2002, postoperative morbidity following RRP was evaluated. After LA, which they performed in 69.3% of patients, they found significantly higher complication rates. However, during the last some years the view about benefits of pelvic LA has changed. A recent study by Abdollah et al. showed a direct link between a higher number of resected lymph nodes and an increased survival rate [42]. The authors showed that tumor-specific metastasis-free survival was significantly increased in patients with more than 14 resected lymph nodes. In our study an average of 18 (CT-group) and 17 (4DF-group) lymph nodes, respectively, were resected, which is higher than the suggested threshold of 14 found by Abdollah et al. [42] for enhanced survival. From the oncological point of view in high risk cancer extensive lymph node dissection is mandatory to ensure best patients survival, despite risk of an increased rate of LC or other complications.

A retrospective multicenter study by Khoder et al. [41] showed that LC with ≥100 mL volume significantly and more frequently provoke complications necessitating interventions like puncture, percutaneous drainage, or surgical therapy such as marsupialization. In our study all LC revealing ≥100 mL volume and sonographic evidence of vascular compression were treated with percutaneous drainage. This was the case in the two 4DF-patients and one individual without polysaccharide treatment. Under this regimen, thromboembolic complications did not occur in our cohort supporting the view that an interventional therapy should be taken into consideration for LC with ≥100 mL volume.

Our strategy in LC treatment followed the suggestion by Kim et al. [43], who described solely percutaneous treatment as simple and safe. In their study drainage-duration was up to 49 days, similar to that in the CT-group of our study. Alternative surgical therapy with marsupialization was offered to our patients with persisting lymphoceles but chosen by none of them. A relapse of LC, as described by Kim et al. [43], was not observed. This might be due to our consequent drainage until secretion had ceased completely.

However, there is no doubt that measures preventing or reducing the incidence of LC in extensive LA would be the first choice if not opposed by concomitant adverse events. In this respect, the results of our study with polysaccharide treatment are promising since the number of late LC in total and the number of LC requiring intervention were half as high in 4DF-patients as compared to controls. The biological processes in the course of polysaccharide degradation might explain this. Poehnert et al. [31] described that the degradation of 4DF occurs via an early foreign body reaction, whereby the polysaccharide particles are surrounded by macrophages and fibroblasts. The immediate presence of these cells is not only helpful for rapid absorption of lymph. It also provides a basis for development of fibrous tissue, which embanks expansion of LC. The instantaneous availability of a network of fibrous tissue might also be causative for the significantly shorter duration of treatment with LC drainage. With drainage times as short as in our polysaccharide treatment group, interventions more invasive than drainage might be considered avoidable. Although the results of our study are promising, they are initial observations and should be confirmed by randomized, multicenter studies in the future.

## 5. Conclusion

In the present study 4DF effectively provided hemostasis especially in treatment of diffuse bleeding in areas hard to approach as well as in areas of the autonomic innervation of the lesser pelvis. The lower total drain loss and significantly reduced incidence of high drain volumes are ascribed to a combined hemo-/lymphostatic effect of the polysaccharide.

Furthermore, in 4DF treated patients incidence of late lymphoceles and lymphoceles requiring treatment was reduced by half. If lymphoceles occurred under polysaccharide treatment, they necessitated a drainage time reduced by more than two-thirds as compared to controls.

These promising observations with 4DF in hemo-/lymphostasis, reduction of incidence, and better treatability of lymphoceles should be confirmed by randomized studies.

## Figures and Tables

**Table 1:** Patient characteristics.

	CT-group	4DF-group	*p* value
Number of patients	51	53	—
Age [years]	67 ± 7.7	67 ± 7.4	0.9030
BMI	27 ± 3.5	27 ± 2.9	0.9176
Removed LN	18 ± 8.3	16.8 ± 8	0.4306
Extended LA	78%	63%	0.1101
Duration of surgical procedure [min]	176 (110–267)	167 (105–286)	0.3122

BMI: body mass index; LN: lymph nodes; LA: lymphadenectomy.

**Table 2:** Gleason-Scores and median PSA-values.

Gleason-Score	CT-group (*n* = 51)	4DF-group (*n* = 53)
3 + 3	4	8
3 + 4	15	13
4 + 3	15	18
4 + 4	9	3
4 + 5	8	7
5 + 4	0	4
5 + 5	0	0
PSA	10.4 ng/mL (3.2–194)	6.7 ng/mL (2.4–138)

**Table 3:** Involvement of lymph nodes and drain loss/LC development.

	CT-group (*n* = 51)	4DF-group (*n* = 53)	*p* value
pN(+)	10 (19.6%)	5 (9.4%)	
Drain-volume pN(+)	280 mL	244 mL	0.750
Drain-volume pN(−)	495 mL	315 mL	0.094
LC in pN(+)	5.9%	5.7%	
LC in pN(−)	31.4%	20.8%	

pN(+): patients with involvement of lymph nodes; pN(−): patients without involvement of lymph nodes.

**Table 4:** Indication for drainage and LC volumes in controls and 4DF-group.

Group	Indication	LC volumes at drainage [mL]
CT-group (*n* = 4)		
Patient 1	Pain, feeling of pressure	300
Patient 2	Vascular compression	110
Patient 3	Leg edema	280
Patient 4	Pain, feeling of pressure	120

		Median: 200 mL

4DF-group (*n* = 2)		
Patient 1	Vascular compression	110
Patient 2	Vascular compression	140

		Median: 120 mL

**Table 5:** Results summary.

	CT-group, *n* = 51	4DF-group, *n* = 53
Drain loss (mean)	452 mL (0–3360 mL)	308 mL (0–950 mL)
Number of patients with drain loss >1000 mL	5	0
Number of early LC	10	11
Number of late LC	16	8
LC with percutaneous drainage	4	2

Median duration of LC drainage	45 days	12 days
Range of duration of LC drainage	23–54 days	10–14 days
